# The Two Isoforms of Lyn Display Different Intramolecular Fuzzy Complexes with the SH3 Domain

**DOI:** 10.3390/molecules23112731

**Published:** 2018-10-23

**Authors:** João M. C. Teixeira, Héctor Fuentes, Stasė Bielskutė, Margarida Gairi, Szymon Żerko, Wiktor Koźmiński, Miquel Pons

**Affiliations:** 1 BioNMR Laboratory, Inorganic and Organic Chemistry Department, University of Barcelona; Baldiri Reixac, 08028 Barcelona, Spain; joaomcteixeira@gmail.com (J.M.C.T.); hctr.fs@gmail.com (H.F.); 2 Institute of Chemical Physics, Vilnius University, Saulétekio al.3, 10257 Vilnius, Lithuania; stase.bielskute@ff.vu.lt; 3 LRB, Scientific and Technologic Centers of the University of Barcelona, Baldiri Reixac, 08028 Barcelona, Spain; mgairi@rmn.ub.edu; 4 Faculty of Chemistry, Biological and Chemical Research Centre, University of Warsaw; Żwirki i Wigury 101, 02-089 Warsaw, Poland; szerko@chem.uw.edu.pl (S.Ż.); kozmin@chem.uw.edu.pl (W.K.)

**Keywords:** Src family kinases, Src, Lyn, SH3 domains, intrinsically disordered proteins, fuzzy complexes, SFKs unique domains, nuclear magnetic resonance, Farseer-NMR

## Abstract

The function of the intrinsically disordered Unique domain of the Src family of tyrosine kinases (SFK), where the largest differences between family members are concentrated, remains poorly understood. Recent studies in c-Src have demonstrated that the Unique region forms transient interactions, described as an intramolecular fuzzy complex, with the SH3 domain and suggested that similar complexes could be formed by other SFKs. Src and Lyn are members of a distinct subfamily of SFKs. Lyn is a key player in the immunologic response and exists in two isoforms originating from alternative splicing in the Unique domain. We have used NMR to compare the intramolecular interactions in the two isoforms and found that the alternatively spliced segment interacts specifically with the so-called RT-loop in the SH3 domain and that this interaction is abolished when a polyproline ligand binds to the SH3 domain. These results support the generality of the fuzzy complex formation in distinct subfamilies of SFKs and its physiological role, as the naturally occurring alternative splicing modulates the interactions in this complex.

## 1. Introduction

The members of the Src family of non-receptor tyrosine kinases (SFK) share a conserved architecture with three folded domains (SH1, which is the kinase domain; and SH2 and SH3 that are regulatory domains) and an N-terminal intrinsically disordered region that includes the SH4 and Unique domains [1]. While the folded domains are highly homologous within the family, the Unique domains (UD) receive this name because they show very distinct sequences and lengths in each member of the family. In contrast to the well-studied region formed by the SH1-SH3 domains, the disordered regions, which are not observed in the crystal structures, remain poorly understood structurally and functionally. The N-terminal SH4 region, which contains myristoyl and palmitoyl groups in most SFKs, acts as a membrane-anchoring site [2]. Recent work has shown that, at least in the case of Src, the leading member of the family, the SH4 region has additional interactions with the SH3 domain that contribute to the stabilization of a fuzzy intramolecular complex involving also the UD [3]. The functional relevance of the UD in Src is dramatically shown by the observation that introducing mutations in specific positions of this disordered region cause a 50% decrease in the invasive capacity of Src-dependent colorectal-cancer cells [3]. The UD of other SFKs with known functional implications include those of Lck (lymphocyte-specific protein tyrosine kinase), directly implicated in the formation of zinc complexes with co-receptors [4]. The UD of Fyn and Lyn mediate the induction of apoptosis [5]. 

Lyn is a member of the SFK mainly, although not exclusively, expressed in hematopoietic cells and affects the immunological response. It plays a key role in the signaling cascade following the recognition of antigens by Ag-specific IgE bound to the subunit of Fc RI expressed in the surface of mast cells. Lyn is associated and phosphorylates specific tyrosine residues in the and units of Fc RI allowing the binding of other SH2 domain containing kinases and triggering mast cell degranulation [6]. Lyn kinase exists as two isoforms, LynA and LynB, generated by alternative splicing and differing by a 21-aa insert in the UD of Lyn A at position 23 [7]. The two isoforms co-immunoprecipitate with Fc RI [6], showed similar levels of total tyrosine and Fc RI phosphorylation when individually expressed in lyn-/- mice but elicited distinct responses in mast cell degranulation [8]. Interestingly, individual expression of the isoforms did not normalize the calcium fluxes and degranulation of lyn-/- mice but the expression of both isoforms normalized the response. Thus, the two isoforms are functionally distinct but the structural origin of the observed differential effects is still missing.

The interplay between intrinsic disorder, post-translational modification and alternative splicing has been suggested to provide physiological plasticity and adaptive responsiveness [9,10,11,12]. We hypothesized that alternative splicing in Lyn may modulate the formation of an intramolecular fuzzy complex involving the UD and SH3 domains of Lyn, analogous to that recently discovered in Src and suggested to be present in other SFKs [3]. We have used nuclear magnetic resonance (NMR) to show the formation of an intramolecular fuzzy complex in LynA and we show that the 21-residue segment (P23 to R43) that is absent in the LynB isoform contains a specific interaction site with the RT-loop of the SH3 domain, thus making the fuzzy complexes formed by the two isoforms, structurally distinct. Natural modulation of the intramolecular fuzzy complex by alternative splicing reinforces its functional significance.

## 2. Results

### 2.1. Protein Constructions and NMR Assignments

**Figure 1:**
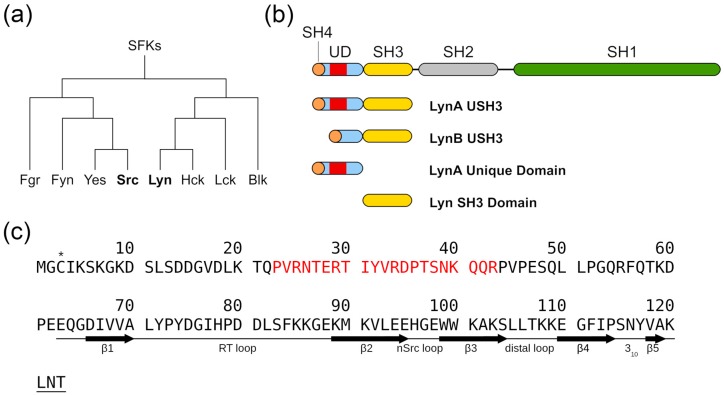
Schematic representation of Lyn structure. (**a**) Cladogram representation of the Src Family Kinases (SFKs) according to its evolutionary proximity. (**b**) Schematic representation of the Lyn protein domains along with the constructs used in the present study. (**c**) Primary sequence of the LynA USH3 sequence. The natural cysteine in position 3, marked (*), was replaced by serine in the constructs used for chemical shift assignments and chemical shift perturbation. The native cysteine 3 was used to attach a nitroxide probe for paramagnetic relaxation enhancement experiments. Residues absent in Lyn isoform B are shown in red. A schematic representation of the experimentally determined secondary structure elements [13] is presented for the residues in the SH3 domain (E^62^-T^123^).

The SFK contains two subfamilies of which Src and Lyn are representative examples (Figure 1a). The SH3 domain of Src was previously shown to form the scaffold of an intramolecular fuzzy complex in which the Unique and SH4 domains were transiently interacting with the loop regions of the SH3 domain, while maintaining their disordered character [3]. We studied protein constructs containing the SH4, UD and SH3 domains (hereafter USH3) of the two Lyn isoforms (A and B) and compared them with constructs containing only the globular (SH3 domain, residues 63–123) or the disordered (SH4-UD residues 1–62) regions (Figure 1b). The sequence of USH3 LynA is shown in Figure 1c. The 21-residue segment (residues 23–43) that is absent in LynB is indicated. For convenience we will use the same numbering in LynB and LynA, thus the residue following Q22 in LynB is P44. The native cysteine in position 3 was replaced by serine in the constructs used for chemical shift perturbations, although the native construct was used to attach a paramagnetic probe in some experiments. The N-terminal region of the SH4 domain had two additional residues (GA) preceding the initial methionine resulting from the protease cleavage used in the purification protocol.

The assignment of the SH3 domain of Lyn had been previously reported [13]. Here we combined the previously published information and a series of 3D BEST-TROSY experiments: HNCO, HNcaCO, HNCA, HNcoCA, HNCACB, HNcoCACB, HNcocaNh recorded using non-uniform sampling and co-processed with qMDD [14] to assign the SH3 domain in the USH3 constructs and in the isolated form under our experimental conditions (phosphate buffer 20 mM, EDTA 0.1 mM, pH 7.5 at 298 K). Assignments were transferred to other temperatures by recording spectra at various temperatures.

The SH4 and UD chemical shifts show the low dispersion typical of disordered regions either alone or when part of the to the USH3 construct. Assignment of the disordered regions was carried out by combining five-dimensional methods: (HACA)CON(CA)CONH [15], HN(CA)CONH [16], HNCOCACB [15], with standard 3D methods (HNCO, HNcaCO, HNCA, HNcoCA, HNCACB, HNcoCACB). The assignment of the disordered regions was done at 278 K. Missing correlations in specific regions of the UD hampered the initial assignment attempts using non-denaturing conditions. A nearly complete backbone assignment could be achieved by recording the spectra of a construct containing only the disordered region of LynA in the presence of 4 M urea. The assignments were transferred to the urea-free conditions by following the chemical shift changes during a urea titration. An overview of the ^1^H-^15^N HSQC spectra of the different domains and constructs with their respective assignment is available in the Supplementary Materials Figures S1–S3.

LynB showed very similar chemical shifts for the residues in the disordered regions that are present in the two isoforms indicating that the common residues experience equivalent environments. LynB constructs were assigned by comparison to LynA.

The backbone NH residues were assigned for 90 out of 95 non-proline residues for Lyn B and 109 residues out of 114 for Lyn A. The missing assignments are the valine residue in the PVP sequence, the two glutamic acid residues followed by a glutamine in the connection between the UD and SH3, and N122 that was assigned in the isolated SH3 domain but is lost in the USH3 constructs.

### 2.2. The Two Isoforms of Lyn form Different Intramolecular Fuzzy Complexes Involving the Unique and SH3 Domains

The presence of fuzzy complexes was assessed by chemical shift perturbation, comparing the environment of backbone NH signals from residues in both the SH3 and disordered domains with those of the respective isolated domains. Significant perturbations far from the connecting residues are indicative of interdomain interactions while the retention of low dispersion in the SH4 and UD confirms that these regions remain disordered in the complex.

**Figure 2:**
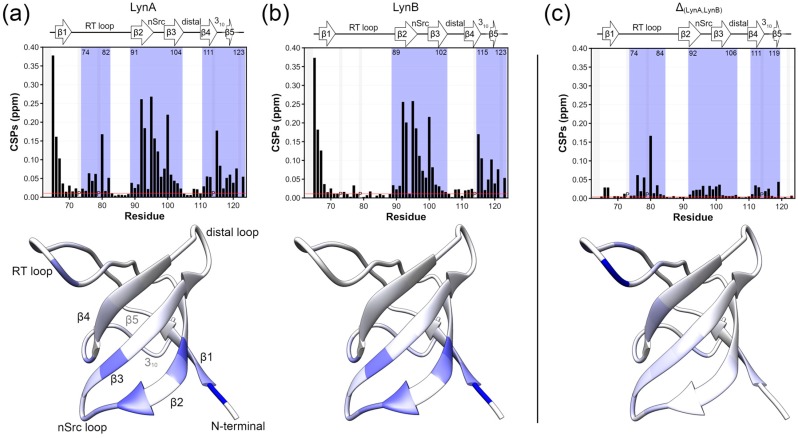
Interaction between the Lyn SH4-Unique Domains and the SH3 domain; for each panel (**top**) ^1^H-^15^N combined chemical shift perturbations (CSPs) in ppm represented in bar plots. Proline residues are identified with “P”. (**bottom**) CSP values are mapped on the structure of the Lyn SH3 domain (PDB 1W1F) using a blue scale ranging from 0 (white) to 0.4 (most intense blue) ppm. (**a**,**b**) observed CSPs in the SH3 domain within the USH3 constructs with respect to the Lyn USH3 isolated domain for LynA and LynB, respectively. Residue numbers in LynB have been modified to match those of LynA. The blue shade in bar plots represents the continuous regions where perturbations are significant, as defined in materials and methods and based on the threshold represented by the red line. The threshold value is the average plus 5 standard deviations of the 10% lowest CSPs (**c**) The difference between CSPs values in (**a**,**b**). The mapping of the differential CSPs (**c**, **bottom**) is in a scale ranging from 0 to 0.15 ppm.

Figure 2a,b show plots of chemical shift perturbations for the SH3 domain in LynA USH3 and LynB USH3, both compared with the isolated SH3 domain. The largest chemical shift perturbations, outside the N-terminal region where the UD is covalently bound are found in the β2 and β3 strands, and the n-Src loop that connects them. Additional perturbations affect the helical turn connecting strands β4 and β5. These regions are similarly perturbed in the two isoforms, although some small differences are observable when Lyn A and LynB are directly compared in Figure 2c. In contrast, the RT-loop is only perturbed in LynA and retains the same chemical shifts of free SH3 in the LynB isoform. Thus, while the two isoforms form similar fuzzy complexes, presumably involving their common regions and the region close to the n-Src loop, the presence of the additional residues in the Unique domain of LynA results in additional interactions with the RT-loop.

**Figure 3:**
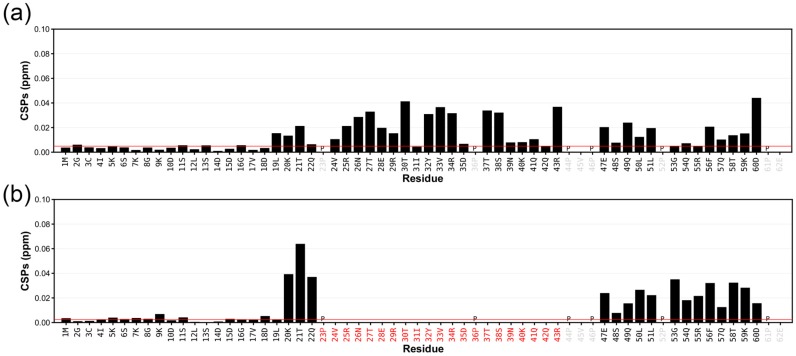
^1^H-^15^N combined chemical shift perturbations (CSPs) in ppm observed between (**a**) the LynA USH3 construct and the isolated LynA SH4-UD construct; (**b**) the LynB USH3 construct and the LynA USH3 construct; in red the residues that are absent in LynB with respect to LynA. For simplicity, the residue numeration for LynB is kept equal to that of LynA. The threshold red line indicates the average plus 5 standard deviations of the CSP values from the 10% least perturbed residues.

The chemical shift differences between LynA SH4-UD domains in the USH3 construct and the SH4-UD construct lacking the SH3 domain are presented in Figure 3a. Interestingly, and in contrast to what had been observed for the fuzzy complex formed by c-Src [3], the initial 18 residues, including the entire SH4 domain, are clearly not affected by the presence of the SH3 domain. Therefore, in the case of Lyn, the fuzzy complex involves only the SH3 and Unique domains. The largest perturbations are observed in the region exclusively present in the LynA isoform (marked). Additional perturbations in the UD are observed in the region between the alternatively spliced segment and the SH3 domain.

Figure 3b compares the chemical shift differences in the SH4-UD regions of the USH3 constructs of LynA and LynB at 278K. Outside the immediate neighborhood of the spliced fragments, perturbations are observed for residues in the UD located between the splicing site and the SH3 domain, while no chemical shift differences are found in the SH4 domain. The observed perturbations together with the small chemical shift differences observed outside the RT-loop between LynA and LynB suggest that the last 16 residues of the UD represent the interaction site with the nSrc and distal loops of the SH3 domain, which is common to the two isoforms but the fuzzy interaction is perturbed by the additional interactions involving residues 23–43 of LynA and the RT-loop. 

Taken together, the chemical shift perturbation experiments show that the alternatively spliced region in LynA is interacting with the RT-loop in the SH3 domain and this is the main differential effect observed between the two isoforms.

### 2.3. The SH4 Domain Also Approaches the SH3 Domain, Although Does Not Show a Direct Interaction

In contrast to chemical shift perturbations, which reflect changes in the local environment of the observed nucleus, paramagnetic relaxation provides evidence for long-range or transient interactions [17]. We used the native cysteine at position 3 in the SH4 domain of Lyn to attach the well-known MTSL (*S*-(1-oxyl-2,2,5,5-tetramethyl-2,5-dihydro-1*H*-pyrrol-3-yl)methylmethanesulfonothioate) paramagnetic probe. Unpaired electrons induce very efficient relaxation of close nuclear spins resulting in line broadening and a decrease in the intensity of the NMR peaks. Paramagnetic Relaxation Enhancement (PRE) was quantified by the ratio between the NMR peak intensities in the paramagnetic and its diamagnetic reference sample, obtained by reducing the paramagnetic probe with ascorbic acid.

**Figure 4:**
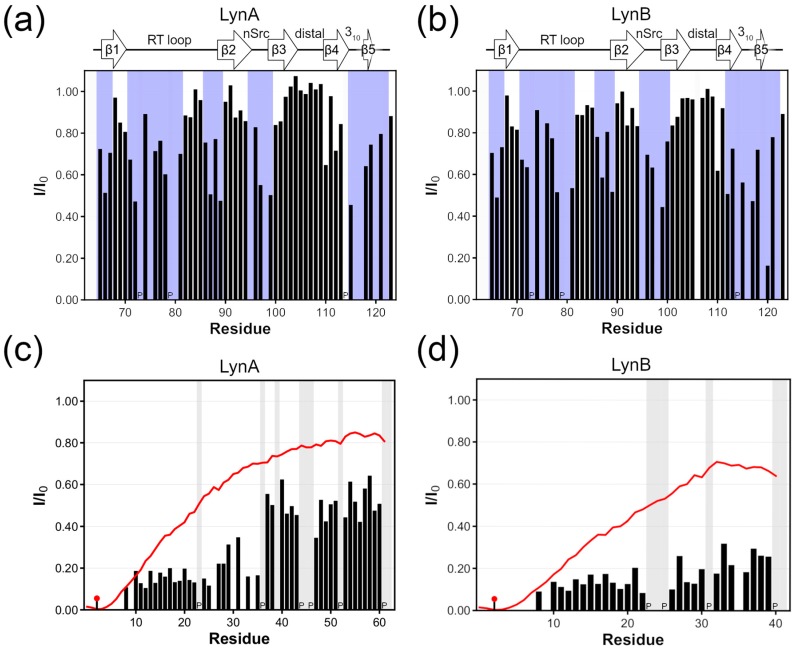
Paramagnetic Relaxation Enhancement (PRE) effects of MTSL paramagnetic probe attached at cysteine 3. (**a**) Intensity ratios observed in the SH3 domain of LynA USH3 construct between the paramagnetic and the diamagnetic sample. (**b**) Same as (**a**) but for LynB USH3 construct. The blue shadow in (**a**,**b**) highlights the continuous sequence regions with I/I_0_ below 0.8 bound by two consecutive residues passing the threshold. (**c**) PRE effects observed within the LynA SH4-Unique domains for the USH3 construct. (**d**) same as (**c**) but for LynB isoform. In (**c**,**d**) a pin marks the position of the tag; in red the theoretical PRE profile considering the SH4-Unique as a random coil. Proline residues are identified with a letter “P”.

Figure 4a,b compares the PRE measured on the SH3 domain in LynA and LynB USH3 with a paramagnetic MTSL probe at position 3. The two isoforms show very similar PRE profiles. The most affected regions correspond to the RT and nSrc surface loops but there is also an effect at the interface between sheets β4 and β5. There are no significant differences between the measured PRE profiles for LynA and LynB, which suggests that the non-interacting SH4 domain is approaching the SH3 surface similarly in the two isoforms and that such proximity is not dependent on the additional 21 residues of the isoform A.

The PREs observed on residues located on the disordered region are affected by the mutual dynamics of the paramagnetic site and the relaxing nuclei. As shown before [3], the random coil model of an intrinsically disordered region is a useful reference to evaluate the extent of compaction throughout PRE observation. Figure 4c,d compares the observed PRE with the predictions of a random coil model (red line) for the flexible region (excluding the SH3 domain). The observed PRE are stronger than expected for a non-interacting random coil, thus indicating a compaction of the flexible region, which can be taken as an evidence for the presence of a fuzzy complex also in Lyn.

### 2.4. A Polyproline Peptide Binding to the SH3 Domain Prevents the Specific Interaction of LynA UD with the SH3 Domain

The canonical regulation of SFKs via the SH3 domain is based on its interaction with the linker connecting the SH2 and kinase domain that contributes to maintain the kinases in a closed, inactive form [18]. Displacement of the linker by external polyproline ligands can lead to kinase activation.

The VSL12 peptide (Ac-VSLARRPLPPLP-OH) [19] is a designed high-affinity ligand for the SH3 domain of various SFK and has been previously used to assess the activation of full-length kinases by displacement of the interaction of the SH3 domain. The K_D_ of VSL12 for the SH3 of Src and Lyn are 4 × 10^−6^ M and 5 × 10^−7^ M respectively, as measured by surface plasmon resonance [20].

**Figure 5:**
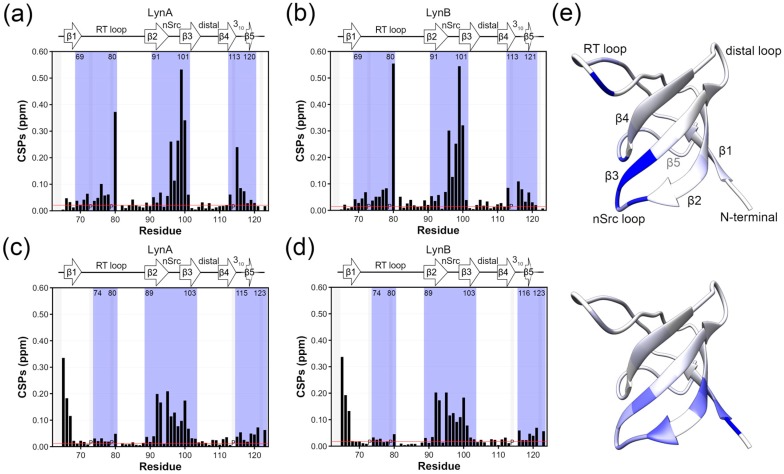
^1^H-^15^N combined chemical shift perturbations (CSPs) in the SH3 domain observed: (**a**,**b**) before and after the addition of 1 equivalent of VSL12 peptide to (**a**) LynA (**b**) LynB USH3 construct; (**c**,**d**) between the SH3 domain of LynA and LynB USH3 and the isolated Lyn SH3 domain, both in the presence of 1 equivalent of VSL12 peptide. The blue shade identifies relevant continuous perturbed regions as defined in the text based on the threshold calculated as the average plus 5 standard deviations from the 10% least affected residues. The letter “P” identifies proline residues. (**e**) CSP values are mapped on the structure of Lyn SH3 (PDB 1W1F) in a blue scale ranging from 0 (white) to 0.3 (intense blue) ppm obtained (**top**) in panel (**a**) (**bottom**) in panel (**c**). The numbering used for LynB is the same as for LynA.

Figure 5a,b shows the CSP induced by the addition of the VSL12 peptide to LynA and LynB USH3. Peptide binding induces changes in the same regions in the two isoforms although the perturbations are quantitatively slightly different. The main differences are observed in the RT loop and, to a lower extent in the distal loop. In order to see if the interaction between the disordered region and the SH3 domain was maintained in the presence of a tightly bound ligand, we compared the chemical shifts of USH3 and isolated SH3 constructs in the presence of an excess VSL12 peptide. The results are shown in Figure 5c,d. Clearly, the presence of the flexible region causes changes in the chemical shift of SH3 residues indicating that the interaction is maintained even when a polyproline ligand is bound to the SH3 domain. However, the difference between LynA and LynB, which involved only the RT-loop is abolished.

## 3. Discussion

All members of the SFK share an intrinsically disordered region with very low sequence homology that contrasts with the high conservation of the kinase and classical regulatory domains SH2 and SH3. Recently, the Unique domain of Src has been shown to be part of a fuzzy complex, involving the SH3 domain and to play an active role in the control by Src of the invasiveness of colorectal cancer cells [3]. The fuzzy intramolecular interaction between the disordered region and the regulatory domain combines the high responsiveness of intrinsically disordered regions with the integrative potential of globular domains. The SH3 domain, thus, appears as a scaffold connecting the disordered and ordered regions in Src.

In this paper we have addressed the question of the generality of this connection by studying Lyn, a SFK from another subfamily, distinct from the one to which Src belongs to. The choice of Lyn was also motivated by the fact that Lyn presents two natural isoforms that differ exclusively in a 21-residue fragment in the Unique domain and originate from alternative splicing. Therefore, the two isoforms may present alternative fuzzy complexes supporting the view that they represent physiologically important regulatory elements that can be tuned by alternative splicing.

We used two NMR approaches to identify the fuzzy complexes in the two Lyn isoforms. Chemical shift perturbations are sensitive to the variations in the environment of individual NH groups when the individual domains are in the complex, as compared to the isolated component. The second approach was Paramagnetic Relaxation Enhancement that is sensitive to long range and transient interactions.

Chemical shift perturbations clearly show that the additional 21-residue segment in LynA is interacting specifically with the RT-loop of the SH3 domain while the following UD residues, which are common in LynA and LynB, are interacting with other regions of the SH3 domain. The LynA-specific interaction can be abolished when a high-affinity polyproline ligand is bound to the SH3 domain, suggesting that the interaction only occurs in the activated form of Lyn, when the SH3 domain is released from the closed, inactive form.

The SH4 domain, which is not making direct contacts with the SH3 domain, according to chemical shift perturbations, is however located close to it, as seen from PRE from the 3-position. The PRE effects on the disordered region (SH4 and UD), when compared to the expectations from a random-coil model, show substantial compaction, compatible with an intramolecular fuzzy complex being present in the two isoforms of Lyn. Taken together, the evidence presented here support a general universal mechanism in which the Unique domain of the various SFK acts as a reader of the intracellular environment, by using the exquisite plasticity of the disordered regions, and transmit this information to generate the proper kinase response, through the fuzzy complex with the SH3 domain [21]. The vast differences in the UD of the various SFK reflect their completely different activities, in spite of having very similar architectures and kinase domains. Posttranslational modifications offer a way of modulating the UD activity in SFK [9,10,22]. Alternative splicing, found in Lyn and Hck [23], can further modulate the UD response.

## 4. Materials and Methods

*Protein Constructs*. *E. coli* optimized protein constructs for C3S LynA and LynB USH3 were bought from ATG: biosynthetics (Merzhausen, Germany) and delivered in pETM-30-X vectors with kanamycin resistance. Proteins were expressed as N-terminal GST fusion proteins with a TEV cleavage site in between. Two non-native residues (GA) remained in the N-terminus after protease cleavage.

*Protein Expression and Purification*. All protein constructs were expressed in the same conditions and in *E. coli* BL21DE3 strain. Overnight pre-culture in LB rich medium was followed by cell growth at 37 °C in 1 L flasks (Rich or Mimimal Media) up to optical density of 0.6–0.8. Protein expression was induced via 1 mM IPTG during 14–16 h at 25 °C. Minimal media contained (/L): 12.8 g Na_2_HPO_4_·7H_2_O, 3 g KH_2_PO_4_, 0.5 g NaCl, 2 mM MgSO_4_, 10 mL of Kao and Michayluk Vitamin Solution 100× (SigmaAldrich, K3129 Sigma, St. Louis, MO, USA), 5 mg/L trace metal cocktail [6 g FeSO_4_·(7H_2_O), 6 g CaCl_2_·(2H_2_O), 1.2 g MnCl_2_·(4H_2_O), 0.8 g CoCl_2_·(6H_2_O), 0.7 g ZnSO_4_·(7H_2_O), 0.3 g CuCl_2_·(2H_2_O), 0.02 g H_3_BO_4_, 0.25 g (NH_4_)6Mo_7_O_24_·(4H_2_O), 5 g EDTA], 3 g *d*-Glucose ^12^C (2 g *d*-Glucose ^13^C), 1 g NH_4_Cl ^14^N (0.5 g NH_4_Cl ^15^N). Cells were harvested via centrifugation for 15 min at 4000 *g* and the pellet recovered in 50 mM Tris buffer, pH 8.0, 300 mM NaCl, 2% glycerol and 1 mM DTT. Cells were lysated with ultra-sounds and insoluble cell debris were removed by centrifugation for 20 min at 75,000× *g*. (H_6_)-GST-Lyn construct was purified from the supernatant via affinity chromatography (HisTrap HP 1 mL, GE Healthcare, Chicago, IL, USA). Imidazole excess from the eluted fraction was removed using PD10 desalting columns (GE Healthcare, Chicago, IL, USA). TEV cleavage was performed O/N at 4 °C or during 4 h at RT, shaking mildly. An additional Nickel affinity purification was performed to separate the Lyn construct from H_6_-GST and H_6_-TEV. The Lyn fraction was further purified using size exclusion chromatography in 20 mM sodium phosphate, pH 7.5, 0.1 mM EDTA. Purification of isolated disordered constructs (SH4-UD) was performed the same way but size exclusion was performed priorly to cleavage by TEV protease.

*S3C mutants for PRE*. Lyn serine 3 was reverted to the WT cysteine to allow functionalization with MTSL tag. Complementary primers used: CAG GGC GCC ATG GGT TGT ATC AAG AGC AAG GGC AAG G and C CTT GCC CTT GCT CTT GAT ACA ACC CAT GGC GCC CTG. Cysteine-containing constructs were purified as described but maintaining 1 mM DTT throughout the whole purification process. The purified Lyn was treated with 5 mM DTT and DTT was removed with PD10 desalting columns, collecting the clean fractions in ice conditions and 16-fold excess of MTSL tag (ChemCruz sc-208677, Dallas, TX, USA) was added immediately after. Overnight stirring at 4 °C produced 100% functionalized protein. MTSL excess was removed by an additional PD10 step.

*NMR spectroscopy*. For backbone assignment of the SH3 domain, BEST-TROSY and Non-uniform sampling (NUS) schemes of HNCO, HNcaCO, HNCA, HNcoCA, HNCACB, HNcoCACB, HNcocacbNH were used in ^13^C-^15^N enriched samples; for CSPs and PREs, ^1^H-^15^N-BEST-TROSY spectra were used on ^15^N-labeled samples. These experiments were recorded in sodium phosphate buffer 20 mM, pH 7.5, EDTA 0.1 mM, 10% D_2_O in a Bruker 600 MHz Avance III spectrometer (Billerica, MA, USA) equipped with a TCI CryoProbe. Experiments focusing on the disordered regions were measured at 278 K while those aiming to observe the SH3 domain at 298 K. 3D spectra were processed with NMRPipe [24] and qMDD [14] while 2D spectra were processed using Bruker TopSpin 3.2 and all spectra were analyzed with CcpNmr version-2. Assignment of SH4-UD in USH3 construct was performed at 278 K using Bruker 800 MHz Avance III HD spectrometer equipped with a TCI CryoProbe (University of Warsaw). 3D spectra were processed using cleaner3d [25], 5D spectra were processed using cleaner5d [26]. Spectra were analyzed using Sparky [27].

Peaklist datasets were exported from CcpNmr [28] and analyzed with Farseer-NMR [29] to generate the Chemical shift perturbations and PRE profiles and respective plots. Combined chemical shift differences were calculated using the following equation:

$$CSP\left(\mathrm{ppm}\right)=\sqrt{\frac{1}{2}\left[\delta_{H}^{2}+{\left(\alpha \cdot \delta_{N}^{2}\right)}^{2}\right]}$$

where *α* is 0.14, but 0.2 for Gly [30]. The CSPs threshold was calculated as the average plus 5 standard deviations of the 10% least-affected peaks. The perturbed regions were defined as the continuous sequence segments containing a majority of peaks exceeding the threshold and delimited by two consecutive residues exceeding the threshold. They are marked with blue shadow in the CSP figures. Paramagnetic relaxation enhancements are given by the peak intensity ratios between the observed condition (paramagnetic) and the reference (diamagnetic). Intensities were calculated as the integral of a fixed box-with of 1 point centered at each peak. Simulated random coil PRE values (theoretical PREs) were calculated from a pool of 10,000 conformers generated using Flexible Meccano [31] as previously described [3,21]. Perturbed regions (shadowed in blue) were defined as continuous sequence segments containing residues with PREs below a 0.8 threshold and delimited by two consecutive residues crossing the same threshold.

*Other software for data representation and figure preparation*. Figures were prepared combining the output from Farseer-NMR [29], UCSF Chimera [32] and GIMP (GNU Image Manipulation Program).

## Supplementary Materials

The following are available online, Figure S1: Assignment of ^1^H-^15^N BEST-TROSY NMR experiments of the SH4-UD domains in USH3 constructs of LynA and LynB isoforms at 278 K, Figure S2: Assignment of ^1^H-^15^N BEST-TROSY NMR experiments of the SH3 in USH3 constructs of LynA and LynB isoforms at 298 K, Figure S3: Assignment of ^1^H-^15^N BEST-TROSY NMR experiments of the isolated SH4-UD domains of LynA at 278 K, Figure S4: PRE effects observed within the Lyn SH4-Unique domains for the USH3 construct.
