# Supplementary material for: The Two Isoforms of Lyn Display Different Intramolecular Fuzzy Complexes with the SH3 Domain
Source: Molecules. 2018 Oct 23;23(11):2731. doi: 10.3390/molecules23112731 (PMC6278449; doi:10.3390/molecules23112731)
Supplement: Supplementary file 1 [file molecules-23-02731-s001.zip › Supplementary Files/20181019_SupplementaryMaterial_.pdf]

molecules

MDPI

SUPPLEMENTARY MATERIAL

The two isoforms of Lyn display different intramolecular fuzzy complexes with the SH3 domain

João M.C. Teixeira[1], Héctor Fuentes [1], Stase Bielskute [2] , Margarida Gairi[3], Szymon Żerko[4], Wiktor Koźmiński[4] and Miquel Pons [1*]

1 BioNMR Laboratory, Inorganic and Organic Chemistry Department, Universitat de Barcelona
2 Institute of Chemical Physics, Vilnius University, Saulėtekio al.3, Vilnius 10257, Lithuania
3 Unitat de RMN d'Alt, Centres Científico-Tecnològics de la Universitat de Barcelona
4 Faculty of Chemistry, Biological and Chemical Research Centre, University of Warsaw

* Correspondence: mpons@ub.edu; Tel.: +34934034683

SUPPLEMENTARY MATERIAL

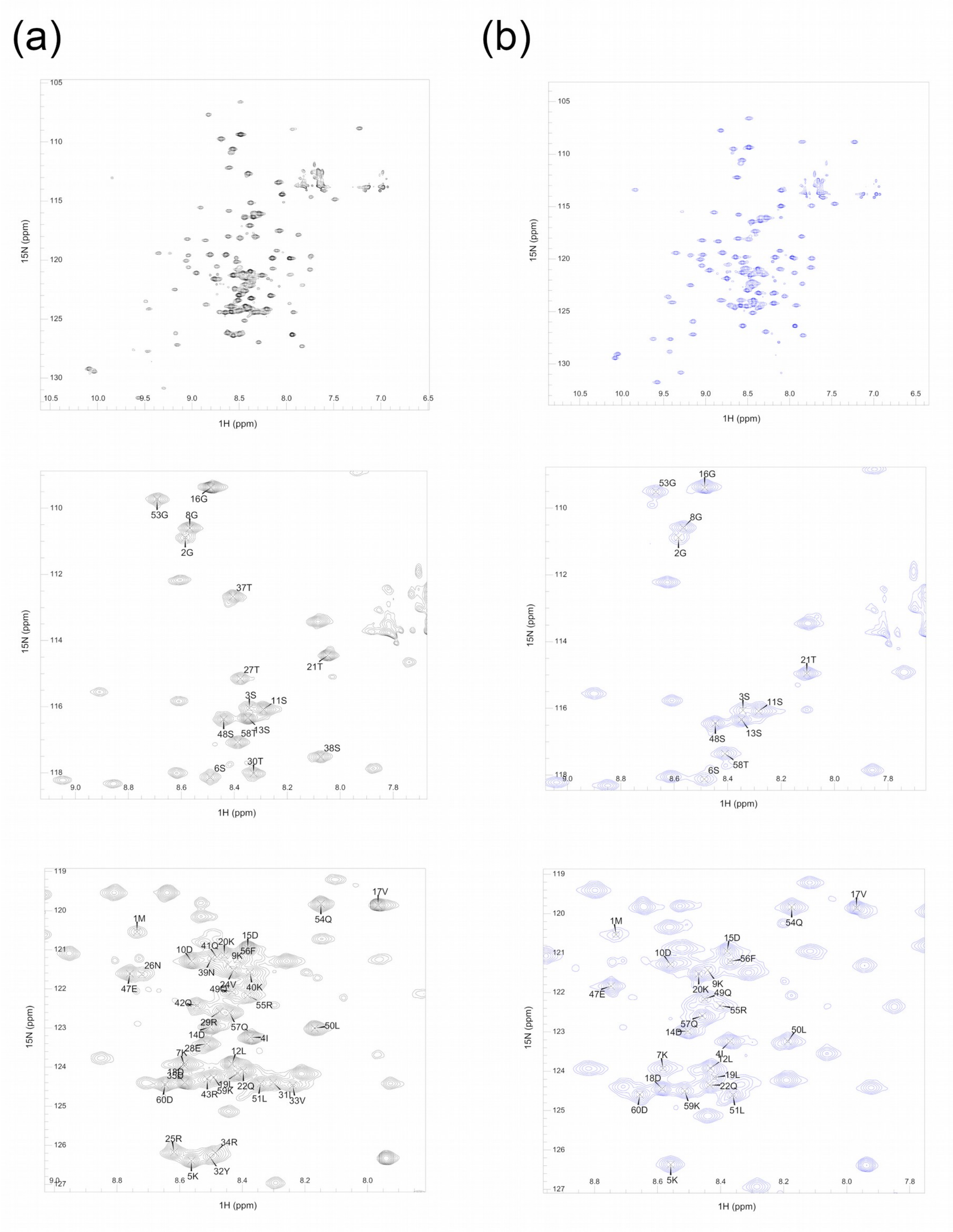

Supplementary Figure 1: ^{1}H-^{15}N BEST-TROSY NMR experiments of **(a)** LynA USH3 and **(b)** LynB USH3; at 278K. Zoomed regions aid on the visualization of the peak assignment labels, only residues from SH4-Unique domain are labelled.

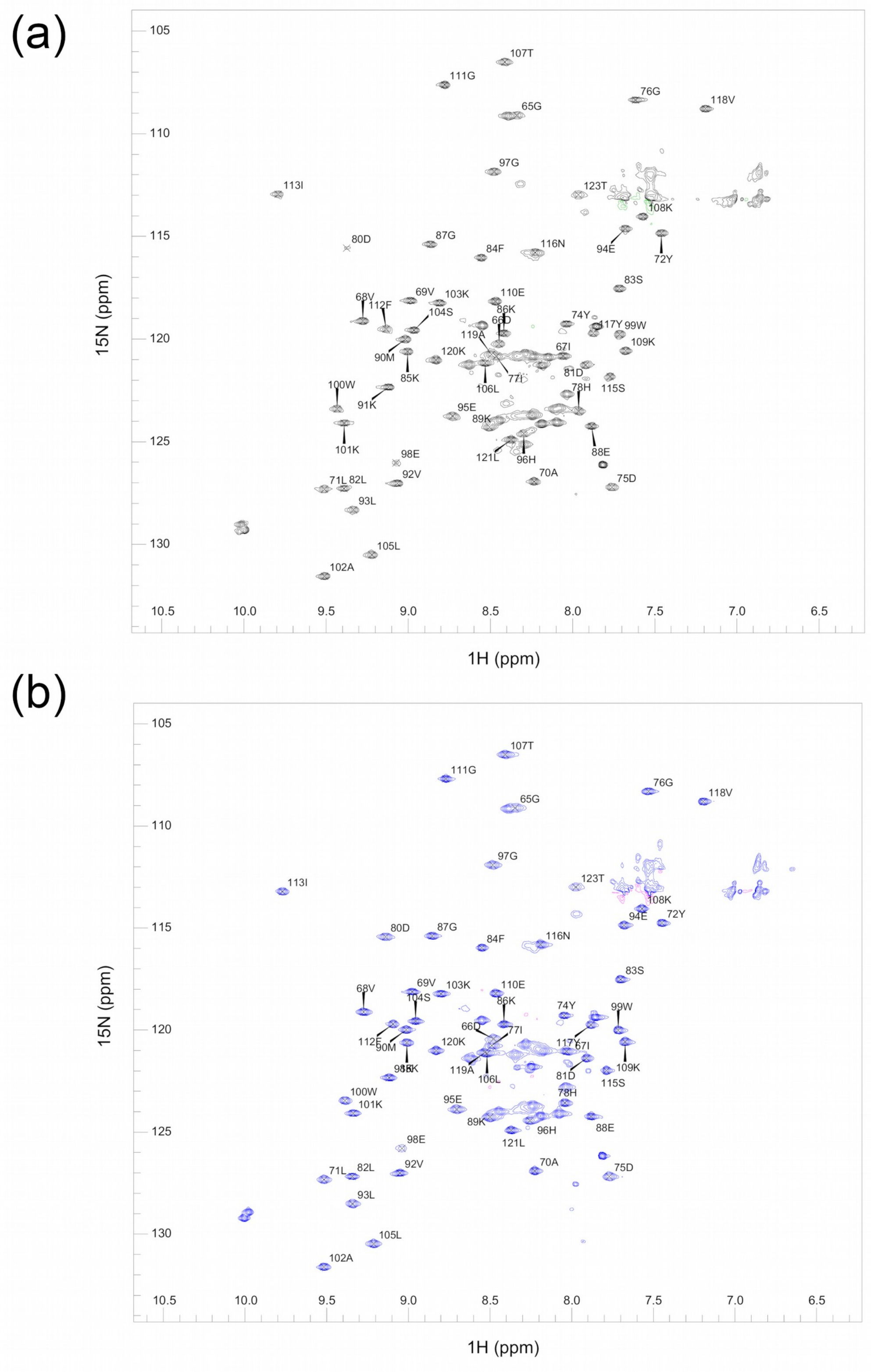

Supplementary Figure 2: ^{1}H-^{15}N BEST-TROSY NMR experiments of **(a)** LynA USH3 and **(b)** LynB USH3; at 298K. Only residues of the SH3 domain are labelled.

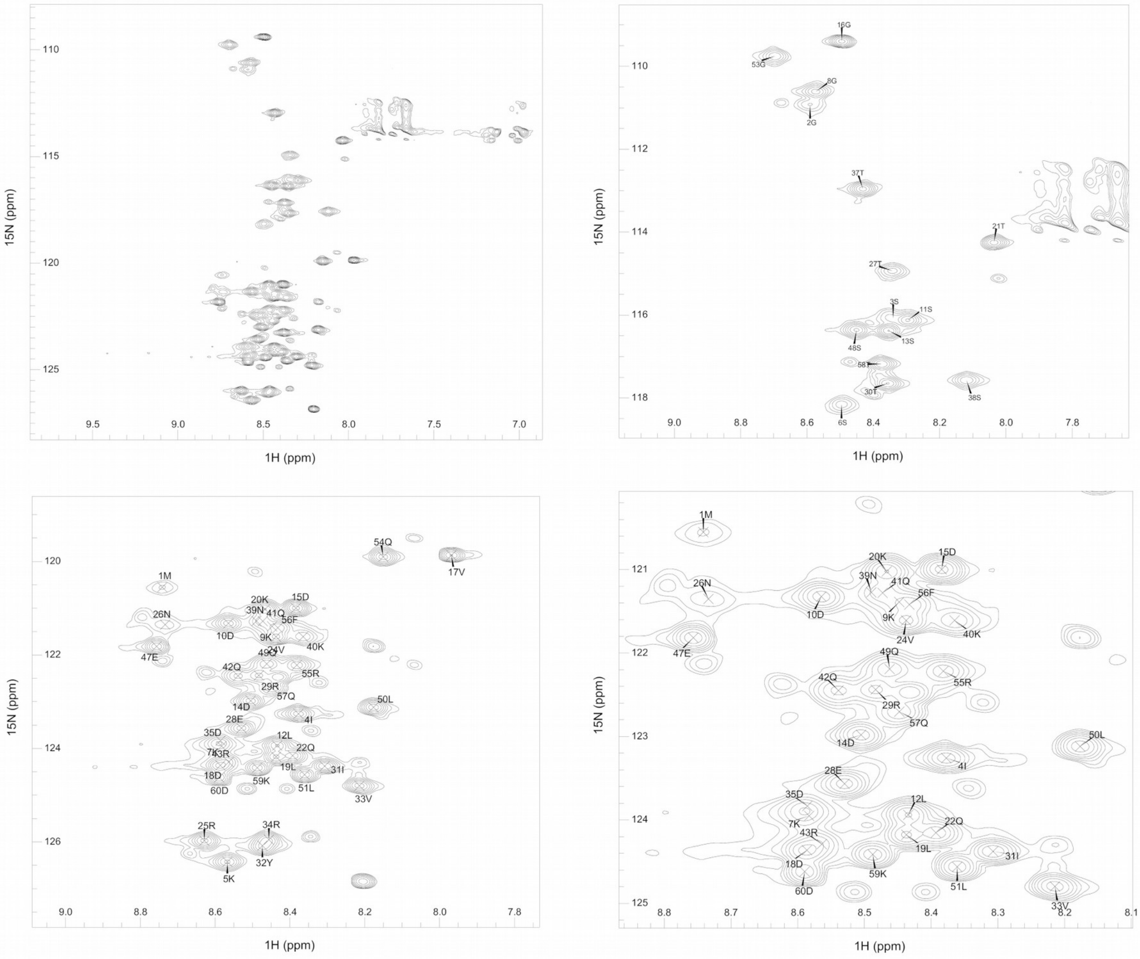

Supplementary Figure 3: ^{1}H-^{15}N BEST-TROSY NMR experiments of Lyn A SH4-Unque Domain isolated construct at 278K. Consecutive zooms amplify the details in the spectra; peaks are labeled according to the obtained assigned.

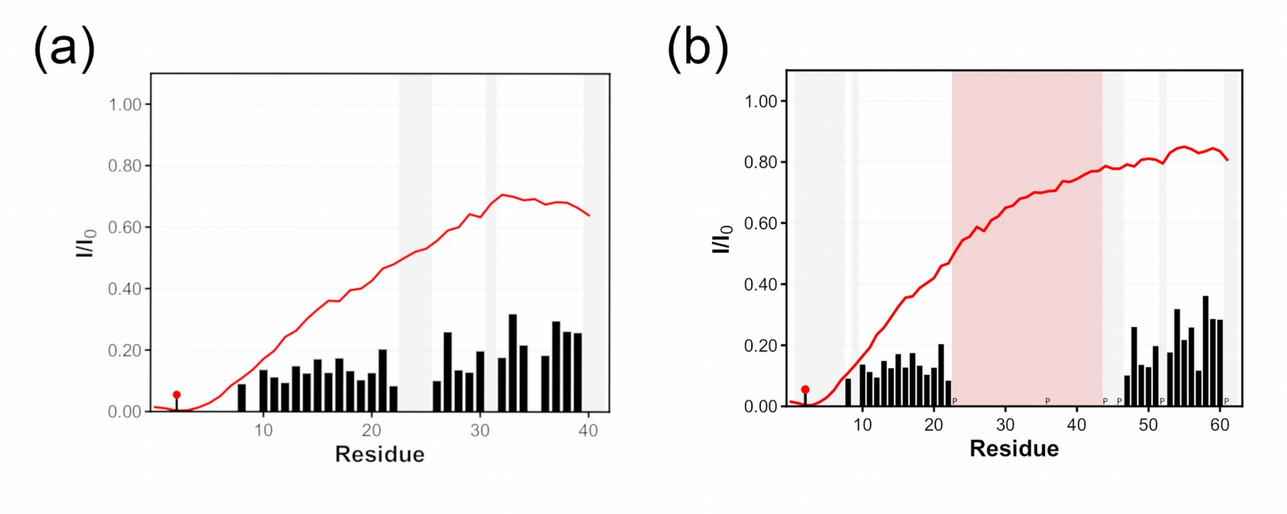

Supplementary Figure 4: PRE effects observed within the Lyn SH4-Unique domains for the USH3 construct. **(a)** for LynB with representation of the theoretical PRE profile considering a random coil model. **(b)** for LynB but with the residue numeration equal to LynA – red shade denotes the residues missing in LynB with respecto to LynA; the red line denotes the theoretical PRE profile as calculated for LynA considering a random coil model.
